# High-Resolution Mass Spectrometry Identification and Characterization of Flavonoids from *Fridericia chica* Leaves Extract with Anti-Arbovirus Activity

**DOI:** 10.3390/molecules27186043

**Published:** 2022-09-16

**Authors:** Ana Flávia Gomes da Cruz, Adriana Cotta Cardoso Reis, Jordano Augusto Carvalho Sousa, Luana Beatriz Araújo Vaz, Breno de Mello Silva, Cíntia Lopes de Brito Magalhães, Markus Kohlhoff, Alaíde Braga de Oliveira, Geraldo Célio Brandão

**Affiliations:** 1 Departamento de Farmácia, Escola de Farmácia, Universidade Federal de Ouro Preto, Campus Morro do Cruzeiro, Ouro Preto 35.400-000, Minas Gerais, Brazil; 2 Departamento de Ciências Biológicas, ICEB, Universidade Federal de Ouro Preto, Campus Morro do Cruzeiro, Ouro Preto 35.400-000, Minas Gerais, Brazil; 3 Laboratório de Química de Produtos Naturais Bioativos, Fundação Oswaldo Cruz, Instituto René Rachou, Belo Horizonte 30.190-009, Minas Gerais, Brazil; 4 Departamento de Produtos Farmacêuticos, Faculdade de Farmácia, Universidade Federal de Minas Gerais, Av. Antônio Carlos, 6627, Belo Horizonte 31.270-901, Minas Gerais, Brazil

**Keywords:** *Arrabidaea chica*, Bignoniaceae, flavonoids, anti-dengue activity

## Abstract

Plant extracts are complex mixtures that are difficult to characterize, and mass spectrometry is one of the main techniques currently used in dereplication processes. *Fridericia chica* is a species with medicinal uses in Latin American countries, used in the treatment of inflammatory and infectious diseases. Extracts of this plant species are characterized by the presence of anthocyanidins. In this study, using high-resolution mass spectrometry coupled with liquid chromatography, it was possible to determine the molecular formula of thirty-nine flavonoids. Fragmentation analysis, ultraviolet spectrum and nuclear magnetic resonance data allowed the partial characterization of the structures of these compounds. The spectral dataset allowed the identification of a series of flavones in addition to the desoxyanthocyanidins common in extracts of the species. The occurrence of some of the proposed structures is uncommon in extracts of species of the Bignoniaceae family, and they are reported for the first time in the extract of this species. Quantitative analyses of total flavonoids confirmed the high content of these constituents in the species, with 4.09 ± 0.34 mg/g of dry plant material. The extract under study showed low in vitro cytotoxicity with CC_50_ ≥ 296.7 ± 1.4 µg/mL for Vero, LLC-MK2 and MRC-5 cell lines. In antiviral activity assays, inhibition of the cytopathic effects of Dengue, Zika and Mayaro viruses was observed, with EC_50_ values ranging between 30.1 and 40.9 µg/mL. The best result was observed against the Mayaro virus, with an EC_50_ of 30.1 µg/mL.

## 1. Introduction

*Fridericia chica* (Bonpl.) L.G.Lohmann (Bignoniaceae) is a climbing plant belonging to the Bignonieae tribe, popularly used in Latin American countries in the treatment of infections, diarrhea, anemia, intestinal pain and uterine inflammation and for the healing of skin wounds [1,2]. Traditionally, it is also used in the production of red coloring, due to the presence of the 3-desoxiantocianidinas chemical constituents characteristic of this species [1].

The presence of flavonoids is common in extracts of this species. Chapman and collaborators were the first researchers to study the leaves of *F. chica*, isolating two 3-desoxyanthocyanidins pigments, carajurin and carajurone, whose structure was fully elucidated by Zorn and collaborators [1,2]. As well as carajurin and carajurone anthocyanidins, Zorn and collaborators isolated 6,7,3′,4′-tetrahydroxy-5-methoxy-flavylium and 6,7,3′-trihydroxy-5,4′-dimethoxy-flavylium [2]. Takemura et al. (1993) isolated 4′,5-dihydroxy-7-methoxyflavone from leaves of this species, and subsequently carajuflavone and luteolin [3]. Barbosa and collaborators reported the isolation of 4′-hydroxy-3,7-dimethoxyflavone, kaempferol and vicenin II [4].

The antiviral activity of this species was first reported by Brandão and collaborators, where the extract of this species inhibited the replication of *Vaccinia virus* (VACV WR) [5]. The same study reported the antiviral activities of the ethanol extracts of eight other species of this family. The best results for antiviral activities were obtained for the extracts of leaves of the species *Arrabidaea ternatum* (EC_50_ of 8.1 mg/mL), *Arrabidaea samydoides* (EC_50_ of 37.1 mg/mL) and *Disctella elongata* (EC_50_ of 4.6 mg/mL) against VACV WR [5].

Several methods have been developed to analyze the constituents in various matrices. Thin-layer chromatography, gas chromatography, high-performance liquid chromatography (HPLC), HPLC–mass spectrometry and capillary electrophoresis (CE) are the most powerful analytical separation methods [6]. In particular, the advent of electrospray ionization (ESI) combined with tandem mass spectrometry (MS/MS) has permitted ready study of the flavonoids and their ion chemistry, and the determination of flavonoids in low concentrations in several extracts [7]. Furthermore, liquid chromatography (LC)-MS techniques are able to separate other single components in complex mixtures and to perform identification and quantification [7].

The compounds were qualitatively identified in the *F. chica* extracts without purification or isolation, and assignment was based on their liquid chromatography (LC)–MS^n^ behavior. Additionally, the quantification of total flavonoids was performed using UV methods, and partial structures were determined using nuclear magnetic resonance (NMR).

## 2. Results and Discussion

### 2.1. Characterization by LC-DAD-ESI-MS and UPLC-HRMS

The UPLC/HRMS results allowed the detection of thirty-nine flavonoids, whose elemental compositions were assigned based on the accurate mass measurements (<5 ppm) provided by the TOF analyzer [8].

After analysis of these data, it was possible to propose the molecular formula and the partial chemical structure for thirty-nine flavonoid compounds detected in their protonated forms, as shown in Table 1. A maximum error of 5 ppm between the experimental and theoretical exact masses of compounds and their proposed structures was observed.

The chromatographic analyses using UV detectors and the mass spectra provided a set of data used for the partial characterization of the compounds present in the extract of this plant species. Among the data obtained, we can highlight the characteristic bands of flavonoids in the UV–visible, the high-resolution mass values, the presence and/or absence of aglycone fragments, the molecular weight of aglycones, the fragmentation data obtained from MS^2^ analysis and the retention times. In addition, comparisons were made with data from isolated compounds and data from the literature [9,10,11,12]. These parameters allowed the separation of the thirty-nine compounds identified into three groups: desoxyanthocyanidins, *C*-glycosylflavones and *O*-glycosylflavones.

**Figure 1:**
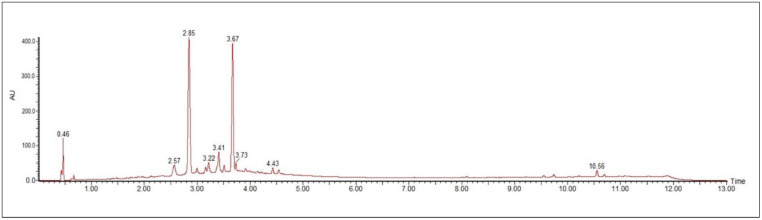
RP-UPLC-DAD profile of ethanolic extract of *A. chica* leaves. Conditions: CHS130 100 RP-18 column (1.7 μm, 50 × 3 mm i.d.). Elution was carried out with a linear gradient of water with 0.1% formic acid and acetonitrile with 0.1% formic acid (from 5% to 95% of acetonitrile with 0.1% formic acid in 11 min), and the UPLC fingerprints were registered on an ACQUITY (Waters) apparatus with a UV-DAD detector (Waters 2996).

**Figure 2:**
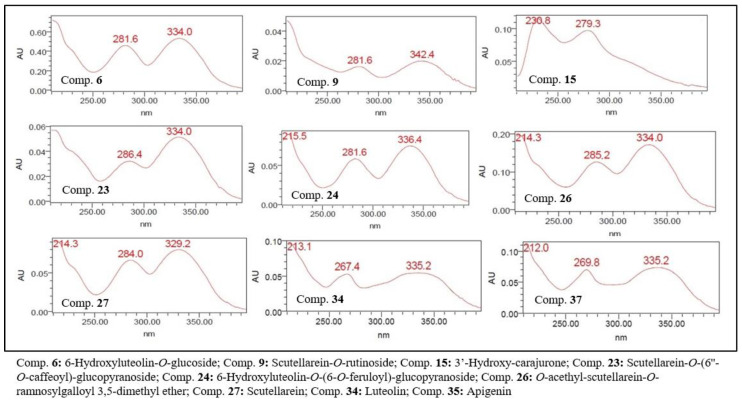
UV spectra (200–400 nm) of flavonoids present in the ethanolic extract of *F. chica* leaves.

The UV spectra of flavonoids, with the exception of anthocyanidins, showed a λ_max_ for the chromophore referring to band 2 of below 335 nm, suggesting that these flavonoids are not oxygenated at position 3 (Table 1, Figure 1). The chromatographic profile of the ethanolic extract obtained by RP-UPLC-DAD is shown in Figure 2.

.

**Figure 3:**
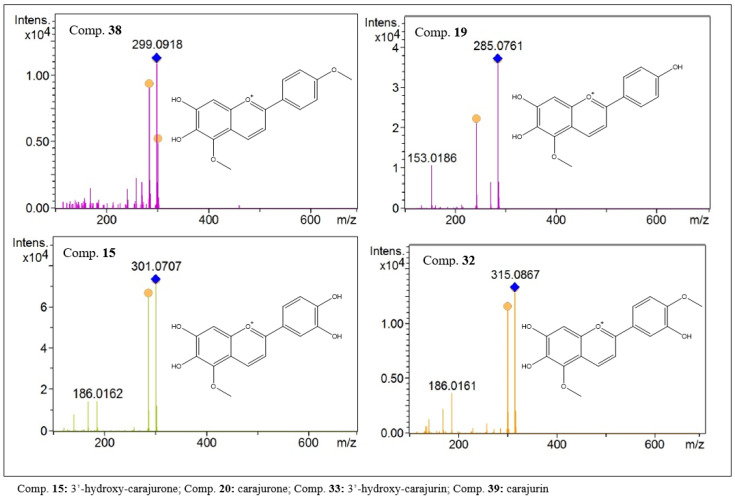
MS^2^ spectra of desoxyanthocyanidins present in the ethanolic extract of *F. chica* leaves.

Desoxyanthocyanidins are widely reported in extracts of this plant, imparting a red color to the extract [1]. In the present study, four anthocyanidins were identified, present as protonated species with [M + H]^+^ *m/z* values of 301.0707, 285.0761, 315.0807 and 299.0918, attributed to the anthocyanidins 3′-hydroxy-carajurone (15), carajurone (19), 3′-hydroxy-carajurin (32) and carajurin (38), respectively. In the fragmentation analysis, losses due to demethylation, decarboxylation and dehydration were observed. Furthermore, fragments resulting from retro-Diels–Alder-type breaks were observed. Fragmentation data for each compound are shown in Table 1 and Figure 3. These anthocyanidins have been previously isolated from leaf extracts of this species [1,2].

**Figure 4:**
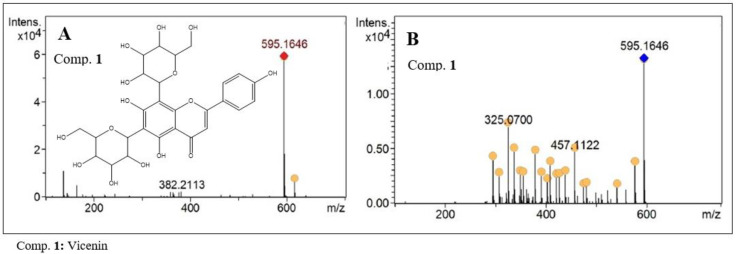
MS^1^ (**A**) and MS^2^ (**B**) spectra of C-glycosyl flavone present in the ethanolic extract of *F. chica* leaves.

C-glycosyl flavones are characterized by the absence of the fragment referring to aglycone flavone when subjected to MS^2^ analysis by electrospray ionization. Compound 1 (C_27_H_30_O_15_) with *m/z* of 595.1646 [M + H]^+^ present in the analyzed extract, was assigned to a flavone of this class (Figure 4). In the MS^2^ analysis, a fragment with *m/z* of 475 was observed, related to a break in the sugar residue. This 120-unit mass loss is characteristic of the presence of a hexose residue in the molecule, with C-C linkage between the aglycone and the corresponding sugar residue [13]. Additionally, losses of H_2_O were observed, which are also quite characteristic in this type of structure. After analyzing the data and comparing it to the literature, the substance Vicenin II was suggested as the likely structure for compound 1 [14]. This compound was isolated from the extract of leaves of this species by Barbosa et al. [9], and *C*-glycosylflavones of similar structure have also been isolated from other species of the Bignoniaceae family [15,16].

The largest number of flavonoids identified in this study belongs to the group of *O*-glycosylflavones and their respective aglycones, with a total of 20 compounds. This group of substances can be divided into five subgroups, according to the aglycone that makes up the main structure. In this work, compounds with aglycone molecular weights of 270, 286, 300, 302 and 316 Da were detected.

**Figure 5:**
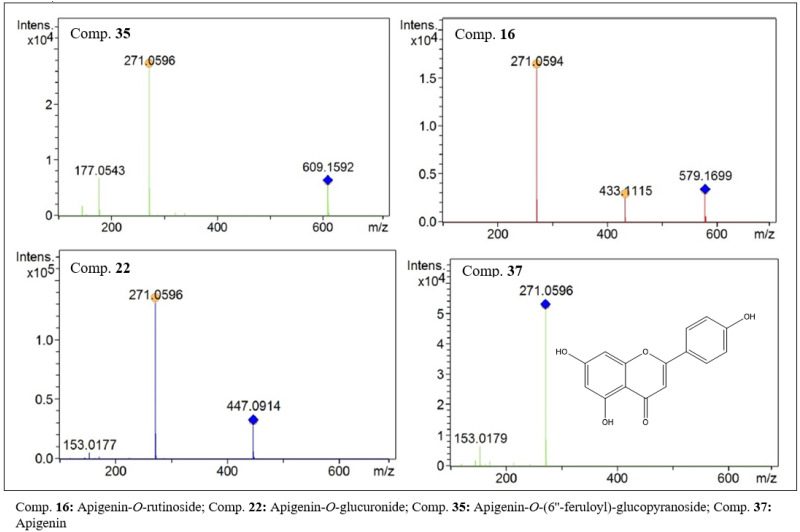
MS^2^ spectra of flavone with apigenin-like aglycones present in the ethanolic extract of *F. chica* leaves.

In the group of flavonoids with an aglycone of 270 Da, apigenin (37, Figure 5) with *m*/*z* 271.0596 [M + H]^+^ and its glycosides derivatives, compounds 16, 18, 22, 35 and 36, were identified.

Compounds 16 (C_27_H_30_O_14_, Figure 5) and 36 (C_30_H_26_O_12_) showed peaks at *m*/*z* values of 579.1699 and 579.1479 [M + H]^+^, respectively. Despite these compounds having similar molar masses, it was possible to differentiate them by their exact masses and also by the retention time (Table 1). Furthermore, in the MS^2^ analysis, compound 16 showed a secondary peak with an *m*/*z* of 433 [M − 146], indicating the loss of a rhamnosyl-type hexose residue. These data suggest that the glycosidic parts of the structures are composed of glucose and rhamnose residues, called rutinoside. Compound 36 presented a fragment with an *m*/*z* of 147 Da, which was attributed to the loss of a *p*-coumaric acid residue. Thus, the flavones apigenin-*O*-rutinoside (16) and apigenin-*O*-(6”-*O-p*-coumaroyl)-glucopyranoside (36) are present in the extract of this species. Compounds 18 (C_21_H_20_O_10_), 22 (C_21_H_18_O_11_, Figure 5) and 35 (C_31_H_28_O_13_, Figure 5) also had a base peak with an *m*/*z* of 271. In the MS^2^ analysis, losses of [M − 162], [M − 176] and [M − 338] were observed, respectively. These results suggest that the losses correlate with glucose, glucuronic acid and a dimer composed of glucose-ferulic acid residues, respectively. Therefore, compounds 18, 22 and 35 were assigned to the derivatives apigenin-*O*-glucoside, apigenin-*O*-glucorinide and apigenin-*O*-(6’’-feruloyl)-glucopyranoside. The presence of cinnamic acid residue contributes to justifying the longer retention time of compound 35 compared to compounds 18 and 22.

In the group whose aglycone has a mass of 286 Da, we have fourteen compounds. In this group, we have compounds derived from the aglycones luteolin (3′,4′,5,7-tetrahydroxyflavone) and scutellarein (4′,5,6,7-tetrahydroxyflavone), which are position isomers. Compounds 28 to 31, 33 and 34 were characterized as being luteolin and its glycoside derivatives. Compounds 4, 9, 10, 12, 14, 23, 26 and 27 were characterized as scutellarein and its glycoside derivatives.

**Figure 6:**
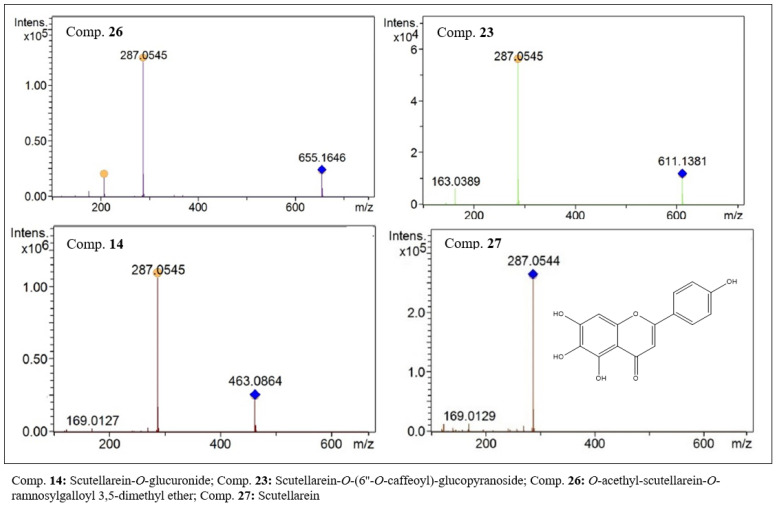
MS^2^ spectra of flavones with scutellarein-like aglycones present in the ethanolic extract of *F. chica* leaves.

In the characterization of compounds derived from scutellarein, it was possible to identify scutellarein (27, C_15_H_10_O_16_, Figure 6) in the form of the aglycone [M + H]^+^ with an *m/z* of 287.0543. Six derivative glycosides were also identified. Compounds with an *m/z* of 611.1380 (23, C_27_H_30_O_16_) in the MS^2^ analysis yielded residues with *m/z* values of 287 and 163. The first derives from the aglycone, and the second was possibly formed from the caffeoyl residue. Therefore, compound 23 (Figure 6) was identified as scutellarein-*O*-(6”-*O*-caffeoyl)-glucopyranoside. At retention times of 14.1 and 14.3 min, two compounds with *m*/*z* values of 463.0860 and 463.0861 were identified. The results of MS^2^ analysis suggest that they are positional isomers. Aglycone residues [M − 176] were observed, which suggests the loss of glucuronic acid residue. Thus, compounds 12 (C_21_H_18_O_12_) and 14 (C_21_H_18_O_12_, Figure 6) were characterized as scutellarein-*O*-glucuronide, but it was not possible to infer the position of the bond of the glucuronic acid residue.

Compounds 4 (C_21_H_18_O_12_), 9 (C_27_H_30_O_15_) and 10 (C_21_H_20_O_11_) showed protonated species with *m/z* values of 463.0881, 595.1644 and 449.1068, respectively, in the first-order analysis. In the MS^2^ analysis of the three compounds, the fragment referring to an aglycone with an *m/z* of 287 Da was observed, which suggests the loss of residues of glucuronic acid [M − 176], of the glucose-rhamnose dimer [M − 308] and of glucose [M − 162]. Thus, it was possible to suggest that compounds 4, 9 and 10 are scutellarein-*O*-glucuronide, scutellarein-*O*-rutinoside and scutellarein-*O*-glucoside, respectively. In this subgroup, a flavone (26, C_32_H_30_O_15_) was also detected that showed a peak of the protonated species with an *m/z* of 655.1643. Despite the high molar mass compared to the other identified flavonoids, this compound eluted with a relatively high retention time, suggesting the presence of low-polarity residues. In the MS^2^ analysis, the aglycone residue with an *m/z* of 287 and a secondary residue with an *m*/*z* of 207 were observed (Figure 6). Data analysis allowed us to suggest the structure of *O*-acetyl-scutellarein-*O*-ramnosylgalloyl 3,5-dimethyl ether. In the library searches for similar chemical structures, flavonoids with this structure were not found, suggesting that it is a new flavone.

**Figure 7:**
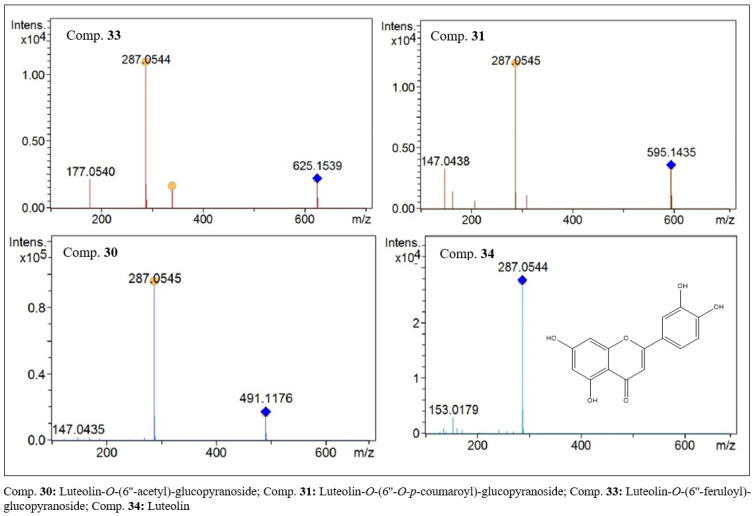
MS^2^ spectra of flavones with luteolin-like aglycones present in the ethanolic extract of *F. chica* leaves.

Luteolin (34, C_15_H_10_O_6_, Figure 7) and its derivatives, compounds 28, 29, 30, 31 and 33, were detected in the leaf extract of this species, and their structures were partially characterized. All compounds presented, as a base peak, the aglycone residue with an *m/z* of 287 [M + H]^+^ in the MS^2^ analyses. In the first-order analyses, protonated species with *m/z* values of 625.1536, 595.1433, 491.1173, 595.1431 and 625.1536 [M + H]^+^ were observed for compounds 28 (C_31_H_28_O_14_), 29 (C_30_H_26_O_13_), 30 (C_23_H_22_O_12_, Figure 7), 31 (C_30_H_26_O_13_, Figure 7) and 33 (C_31_H_28_O_14_, Figure 7), respectively. Thus, we have two pairs of isomers, compounds 28 and 33 with an *m/z* of 625 [M + H]^+^ and compounds 29 and 31 with an *m/z* of 595. In the MS^2^ analysis, losses of [M − 338] and [M − 308] occurred, and fragments with *m/z* values of 177 and 147 were observed, suggesting the presence of ferulic and coumaric acid residues, respectively. Ferulic acid residues were present in compounds 28 and 33, while coumaric acid residues occurred in compounds 29 and 31. Data analysis suggests that the pairs of isomers correspond to the compounds Luteolin-*O*-(6”-feruloyl)-glucopyranoside (28 and 33) and Luteolin-O-(6”-*O-E*-p-coumaroyl)-glucopyranoside (29 and 31). Compound 30, with an *m*/*z* of 491 Da [M + H]^+^ and a base peak with an *m*/*z* of 287 Da, is compatible with the structure of luteolin-*O*-(6”-acetyl)-glucopyranoside.

**Figure 8:**
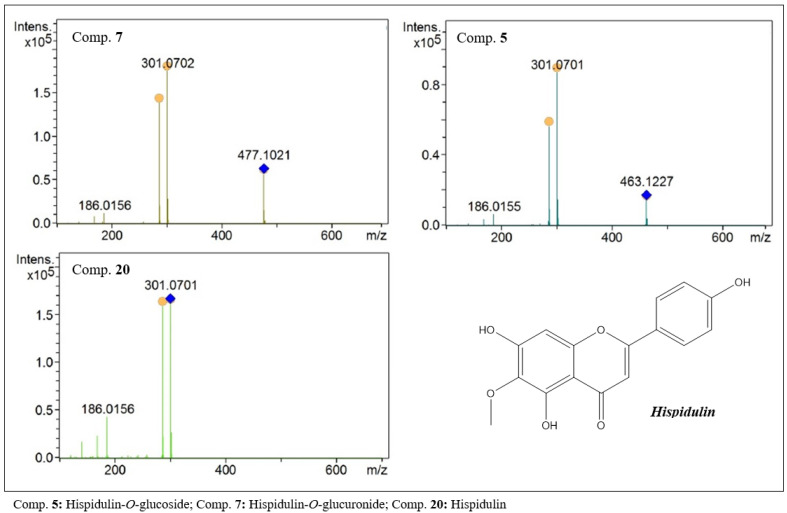
MS^2^ spectra of flavones with hispidulin-like aglycones present in the ethanolic extract of *F. chica* leaves.

**Figure 9:**
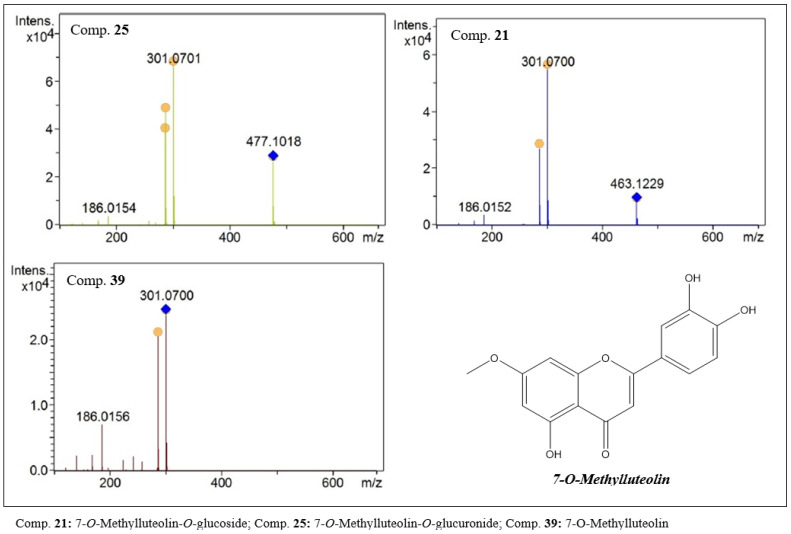
MS^2^ spectra of flavones with 7-*O*-methylluteolin-like aglycones present in the ethanolic extract of *F. chica* leaves.

In the present study, flavones were detected with aglycones of 300 Da. The analysis of the data obtained by LCMS allowed two groups to be suggested. One group consists of hispidulin (20, C_16_H_12_O_6_, Figure 8) and its glycosylated derivatives and the other of 7-*O*-methylluteolin (39, C_16_H_12_O_6_, Figure 9) and derivatives.

Compounds 5 and 7 showed protonated species with *m/z* values of 463.1227 and 477.1019. In the MS^2^ analysis, both compounds showed a base peak with an *m/z* of 301, indicating losses of [M − 162] and [M − 176], respectively. These data suggested the presence of glucose and glucuronic acid residues in the structure of these compounds. As secondary fragments, peaks with an *m/z* of 286 were observed, suggesting the loss of a methyl group from the aglycone. In this way, compounds 5 and 7 were characterized as being hispidulin-*O*-glucoside (5) and hispidulin-*O*-glucuronide (7).

Compounds 21 and 25 are isomers of compounds 5 and 7, respectively. However, they were eluted at longer retention times. These data allow us to suggest structural differences that give compounds 21 and 25 less polarity. In the first-order analysis, they presented *m/z* values of 463.1229 and 477.1017 [M + H]^+^, and in the fragmentation analysis fragments were observed suggesting the presence of glucose and glucuronic acid residues. These compounds were partially characterized as 7-*O*-methylluteolin-*O*-glucoside (21) and 7-*O*-methylluteolin-*O*-glucuronide (25).

**Figure 10:**
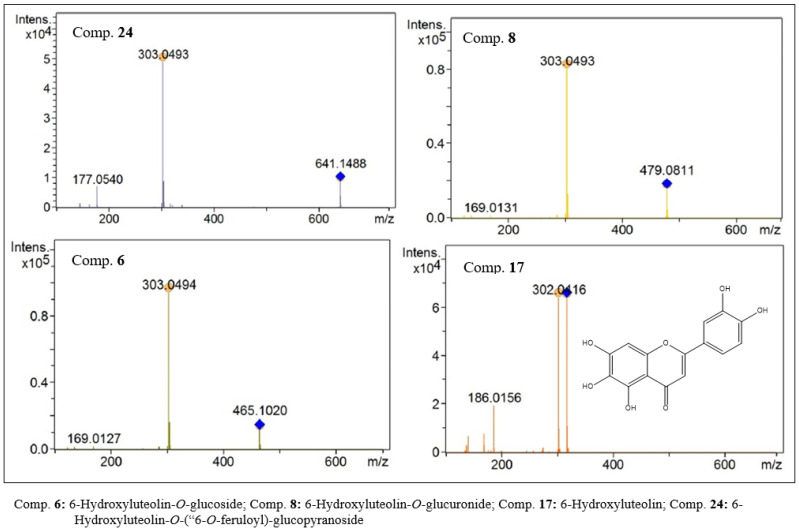
MS^2^ spectra of flavones with 6-hydroxyluteolin-like aglycones present in the ethanolic extract of *F. chica* leaves.

In the group of compounds with a molar mass of 302 Da, five compounds were detected. Compound 17 (C_15_H_10_O_7_, Figure 10) was characterized as 6-hydroxyluteolin. In the MS^1^ analysis, a peak with an *m/z* of 303.0493 was observed, attributed to the protonated species. In the MS^2^ analysis, fragments were observed that corroborate this proposal. Four glycosylated derivatives were also characterized. The data obtained for compounds 8 (C_21_H_18_O_13_, Figure 10) and 13 (C_21_H_18_O_13_) suggest that these compounds are positional isomers. Peaks with *m/z* values of 479.0811 and 479.0809 were observed for compounds 8 and 13. In the MS^2^ analysis, fragments with an *m/z* of 303 were observed as a base peak, suggesting the loss of a glucuronic acid residue [M − 176]. Therefore, compounds 8 and 13 were characterized as being two 6-hydroxyluteolin-*O*-glucuronide derivatives. Two other compounds, 6 (*m/z*: 465.1019, C_21_H_20_O_12_, Figure 10) and 24 (*m/z*: 641.1480, C_27_H_28_O_11_, Figure 10), presented a base peak in the MS^2^ analysis with an *m/z* of 303. As a secondary fragment of compound 24, a peak with an *m/z* of 177 was observed, suggesting the presence of a ferulic acid residue. Data analysis allowed the characterization of compounds 6 and 24 as 6-hydroxyluteolin-*O*-glucoside and 6-hydroxyluteolin-*O*-(6-*O*-feruloyl)-glucopyranoside, respectively.

**Figure 11:**
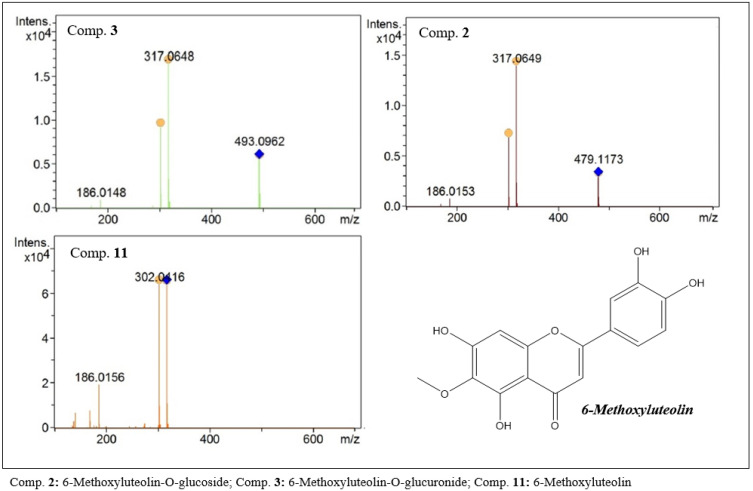
MS^2^ spectra of flavones with methoxy luteolin-like aglycones present in the ethanolic extract of *F. chica* leaves.

In the group of high-polarity flavonoids detected in the extract are the 6-Methoxyluteolin derivatives (11, C_16_H_12_O_7_, Figure 11) represented by compounds 2 (C_22_H_22_O_12_, Figure 11) and 3 (C_22_H_20_O_13_, Figure 11). In the first-order analyses, protonated species with *m/z* values of 479.1173 and 493.0962 were detected. In the fragmentation analysis, a base peak with an *m/z* of 317 was observed, corresponding to a loss of M-162 for compound 2 and M-176 for compound 3. For both compounds, a secondary fragment with an *m/z* of 302 was observed, suggesting the loss of a methyl in both compounds. The data obtained allow us to suggest that they are the flavonoids 6-methoxyluteolin-*O*-glucoside (2) and 6-methoxyluteolin-*O*-glucuronide (3).

Of the thirty-nine compounds characterized, thirty-seven had molar masses and spectral data compatible with known substances. The anthocyanidins present in the studied extract have already been reported in extracts of this species in previous studies [1,2]. Vicenin II, apigenin and luteolin are flavones that have also been isolated from the species [3,4]. Siraichi et al., employing LC–ESI-MS/MS analysis, identified the presence of 6-hydroxyluteolin, hispidulin and scutellarein in the ethanol–water extract of leaves of this species [17]. The other flavonoids characterized here are being reported for the first time in this species. Compounds 24 and 26 are possibly new substances and need isolation to confirm their structures. The data obtained in this work only suggest that they are derived from luteolin and scutellarein.

### 2.2. Quantification of Flavonoids

Flavonoids are polyphenolic compounds commonly found in extracts of this species [3]. Literature data suggest that these compounds are responsible for the biological activities described for extracts of this plant species [18]. Quantification of total flavonoids was performed by UV spectrometry. In these analyses, AlCl_3_ was used as an agent to promote the bathochromic shift. The aluminum cation forms stable complexes with the flavonoids present in the sample, promoting an increase in UV absorption. In the samples in this study, the average concentration of total flavonoids present in the leaves of *F. chica* was 4.09 ± 0.34 mg/g of dry plant material.

### 2.3. Characterization by NMR

**Figure 12:**
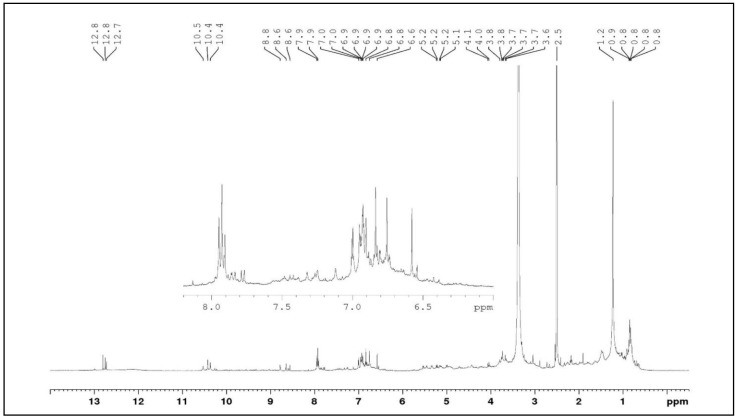
HSCQ contour map of flavonoids present in the ethanolic extract of *F. chica* leaves (^1^H NMR—400 Hz, ^13^C NMR—100 Hz, DMSO-d6, δ).

To contribute to the characterization performed by HRMS, analyses were performed using NMR (H^1^ and HSQC). In the H^1^ spectrum (Figure 12), chemical shift signals (δ) between 12.7 and 13.0 ppm were observed, attributed to hydrogens attached to chelate hydroxyl groups, common in oxygenated flavonoids at position 5. Signals (δ) between 8.5 and 10.5 ppm were attributed to hydrogens present in ortho dihydroxy systems that are present in luteolin-like and scutellarein-like flavonoids. Signals (δ) between 6.5 and 7.8 ppm can also be observed in this spectrum, which were attributed to hydrogens present in aromatic systems. In the lower part of the spectrum (3.5 to 4.0 ppm), signals (δ) compatible with hydrogens present in sugar residues were observed.

**Figure 13:**
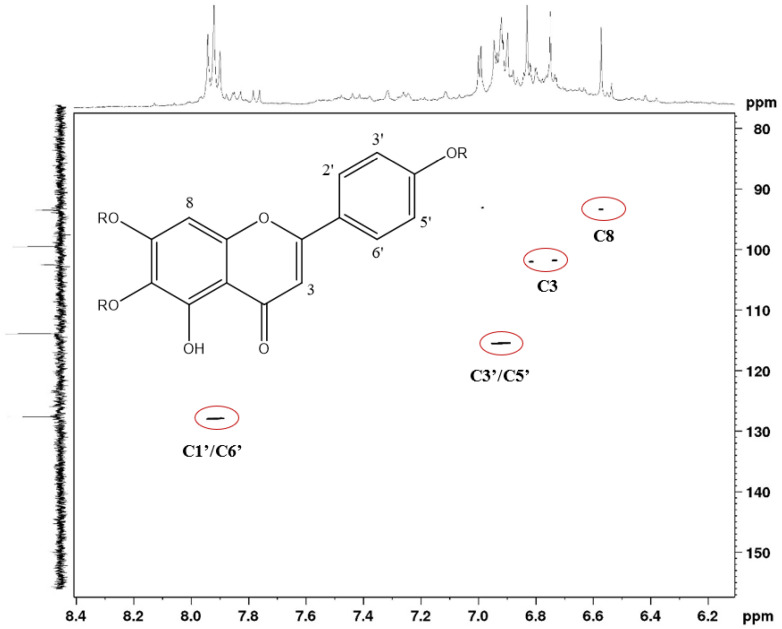
HSCQ contour map of flavonoids present in the ethanolic extract of *F. chica* leaves (^1^H NMR—400 Hz, DMSO-d6, δ).

In the HSQC experiment (Figure 13), it was possible to determine, through heteronuclear coupling (^1^*J*_CH_), the presence of hydrogenated carbons at 129.0 ppm and 115.0 ppm, compatible with a para-disubstituted aromatic system (typical AA’BB’ system) present in apigenin-like and scutellarein-like flavonoids. In addition, it was possible to observe signals (δ) of hydrogenated carbon at approximately 94.0 ppm, which are common in unsubstituted C-8 flavones. Additionally, signals (δ) were observed at approximately 101.0 ppm, which can be attributed to anomeric carbons present in sugar residues, and at 104.0 ppm, related to non-oxygenated carbons in the 3-position of flavones. The spectral data obtained by NMR analyses corroborates the findings in the LCMS analyses.

### 2.4. Cytotoxic Effect and Antiviral Activity

**Figure 14:**
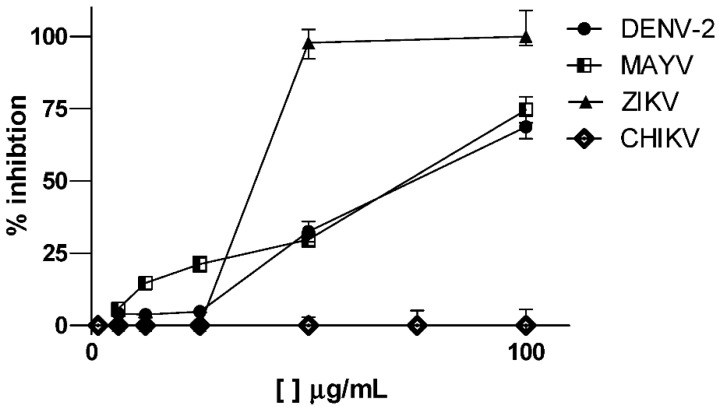
Dose–response curves for antiviral activity of ethanolic extract from Fridericia chica leaves against Dengue (DENV-2), Zika (ZIKV), Mayaro (MAYV) and Chikungunya (CHIKV) viruses.

The cytotoxic effect of the ethanol extract was evaluated in Vero, LLC-MK2 and MRC-5 cell cultures. The CC_50_ values for each cell line are described in Table 2. The extract showed low cytotoxicity, with CC_50_ values ≥ 296.7 µg/mL. Previous studies have already reported low toxicity for extracts of this species [5]. The extract showed antiviral activity against DENV 2, MAYV and ZIKV, with EC_50_ values ranging from 30.1 to 40.9 µg/mL (Figure 14). The best result was obtained against MAYV, with an EC_50_ of 30.1 µg/mL and an IS of >13.3. The antiviral activity of this species against *Herpes simplex virus* type 1 and *Vaccinia virus* has already been reported by Brandão et al. [5].

In addition to the reported antiviral activities, publications have recently shown that *F. chica* is a source of compounds with antiparasitic activity, which once again shows the importance of research with extracts of this species [18,19]. Furthermore, flavonoid compounds present in large amounts in extracts of this species have been shown to be potent inhibitors of the viral replication cycle [20,21,22,23].

## 3. Methods and Materials

### 3.1. Plant Material and Chemicals

The *F. chica* samples were collected in Caratinga, state of Minas Gerais, Brazil. The species was identified by Dr. J. A. Lombardi, Department of Botany, Institute of Biosciences, UNESP, Rio Claro, Brazil. A voucher specimen was deposited in the BHCB/UFMG, Belo Horizonte, Minas Gerais, Brazil, under the number 23859.

### 3.2. Extract Preparation

The leaves (69.0 g) were dried in a ventilated oven at 40 °C for 72 h, ground and exhaustively extracted by percolation with ethanol at room temperature for 48 h each time. A crude dark-green ethanol extract residue (EEFCL, 13.6 g) was obtained in a rotatory evaporator under reduced pressure at 50 °C. The ethanolic extract was subjected to antiviral activity tests.

### 3.3. Liquid Chromatography–Mass Spectrometry in Series (LC-DAD-ESI-MS)

Ethanolic extract from *F. chica* leaves was dissolved in methanol and filtered through a 0.2 µm microfilter. LC-DAD-MS analyses were performed using an ACQUITY UHPLC system (Waters) coupled to UV-DAD and an ion trap mass spectrometer [9]. Chromatographic separation was performed using an ACQUITY UHPLC HSS RP-18 column (1.7 μm, 50 × 2 mm i.d.) (Waters). The mobile phase consisted of water with 0.1% formic acid (solvent A) and acetonitrile with 0.1% formic acid (solvent B). The elution protocol was a 0–11 min linear gradient from 5% to 95% B. The flow rate was 0.3 mL min^−1^, and the sample injection volume was 4.0 μL. The UV spectra were registered from 190 to 400 nm. Ion trap mass spectrometer analyses were performed using the following conditions: positive and negative ion mode; capillary voltage, 3500 V; capillary temperature, 320 °C; source voltage, 5 kV; vaporizer temperature, 320 °C; corona needle current, 5 mA; sheath gas, nitrogen, 27 psi. Analyses were conducted in full scan mode (100–1500 Da) [9].

### 3.4. High-Resolution Mass Spectrometry Analyses

UHPLC-HRMS/MS analyses were performed on a Nexera UHPLC system (Shimadzu, Kyoto, Japan) combined with a maXis high-resolution ESI-QTOF mass spectrometer (Bruker, Billerica, MA, USA) controlled by the Compass 1.7 software package (Bruker). A 5 μg sample was injected into a Shimadzu Shim-Pack XR-ODS-III column (C18, 2.2 µm, 2.0 × 150 mm) at 40 °C, with a flow rate of 400 μL/min. The mobile phases A and B (0.1% formic acid in MilliQ water and acetonitrile, respectively) formed an eluent gradient from an initial 5 min of 5%B to 100% B in 40 min, with a hold at 100% B for 5 min. After UV-PDA detection (190–450 nm), mass spectra were acquired in positive mode at a rate of 5 Hz. Ion-source parameters were set to 500 V end-plate offset, 4500 V capillary voltage, 3.0 bar nebulizer pressure and 8 L/min and 200 °C dry gas flow and temperature, respectively. Data-dependent fragment spectra were recorded using a collision energy range between 15 and 60 eV. Ion cooler settings were optimized for an *m*/*z* 100–1500 range, using a calibrant solution of 1 mM sodium formate in 50% 2-propanol. Mass calibration was achieved by initial ion-source infusion of 20 μL calibrant solution and post-acquisition recalibration of the raw data. Compound detection was achieved by chromatographic peak dissection with subsequent formula determination according to the exact mass and isotope pattern (MS^1^). Putative identification was based on comparison of compound fragment spectra (MS^2^) with reference spectra from an in-house database of standard compounds (FIOCRUZ-Minas), the public spectrum database MassBank and in-silico fragment spectra generated from the Universal Natural Product Database [24,25].

### 3.5. Quantitative Flavonoids Analysis

The measurements were carried out using a UV-M51 spectrophotometer (Bel, Suresnes, France), with a quartz cuvette with a 10 mm optical path (Sigma, St. Louis, MO, USA), heating mantle (Fisatom, São Paulo, Brazil) and industrial mill (Manesco & Ranieri). An analytical balance and glassware were used for all parameters. The content of flavonoids calculated as quercetin in the plant samples was determined by the method of the Brazilian Pharmacopoeia [26].

For the hydrolysis of flavonoids, 100 mg of *F. chica* leaves extract was weighed and transferred to a 50 mL round-bottom flask. Then, 1 mL of 0.5% (*w*/*v*) aqueous methenamine solution, 20 mL of acetone and 2 mL of concentrated chloric acid were added. The mixture was heated under reflux for 30 min. After refluxing, the mixture was left at room temperature for 30 min. The mixture was then filtered into a 50 mL volumetric flask. The residue was washed with 20 mL of acetone. Then, the flask was made up to the required volume with acetone [26]. Analyses were performed in triplicate.

Aliquots (1.0 mL) of the solution from each triplicate were transferred to 25 mL volumetric flasks. Then, 0.6 mL of glacial acetic acid, 10 mL of 20% methanolic pyridine solution and 2.5 mL of a 5% methanolic solution of AlCl_3_ were added to each flask. The contents of each flask were made up to the required volume with distilled water. The triplicates were kept protected from light at room temperature for a period of 30 min. The analyses were performed in a spectrophotometer at 420 nm, using quartz cuvettes with an optical path of 10 mm [26,27].

### 3.6. NMR Spectroscopy

The ^1^H NMR spectra and the two-dimensional HSQC contour maps were obtained in the Multi-user Molecule Characterization Laboratory at the Pharmacy School (UFOP) in Ouro Preto, Brazil. Bruker Ascend^TM^ 400 equipment was used to obtain the spectra. The chemical shifts are given as δ (ppm). A portion of EEFCL (100.0 mg) was dissolved in MeOH/H_2_O (6:4) solution and sequentially partitioned with ethyl acetate. The fraction in ethyl acetate was dried on a rotary evaporator and transferred to amber vials. For the NMR analysis, 30 mg of the fraction in ethyl acetate was solubilized in DMSO*_d6_*.

The ^1^H-NMR spectra were acquired at 400-MHz and a temperature of 298 K, with an Ascend 400 spectrometer (Bruker^®^) using a broadband observation (BBO) probe. The spectra were recorded using a presaturation pulse sequence with a composite pulse (Bruker zgcppr) for water suppression [28]. A total of 128 transients were collected into 64 K data points, with a relaxation delay of 1 s. A spectral width of 8012.82 Hz and an acquisition time per scan of 4.09 s were used. An exponential line-broadening function of 0.3 Hz was applied to the free induction decay (FID) prior to Fourier transformation (FT).

### 3.7. Cell Culture and Virus

Vero cells (kidney cells from African green monkey *Cercopthecus aeothiops*, ATCC CCL-81 ^TM^, Manassas, VA, USA), LLC-MK2 cells (kidney cells from *Macaca mulatta*, monkey, rhesus, ATCC CCL-7™) and MRC-5 cells (fibroblast cells from lung, human, ATCC CCL-171^TM^) were cultured in Dulbecco’s modified Eagle’s medium (DMEM) at 37 °C, in a 5% CO_2_ atmosphere, supplemented with 5% fetal bovine serum (FBS), 100 U/mL penicillin/streptomycin and 5 µg/mL amphotericin B. Zika, Chikungunya and Mayaro viruses were kindly donated by Dra. E. Kroon (UFMG, Belo Horizonte, Brazil), Dr. E. Arruda Neto (UNIFESP, São Paulo, Brazil) and Dr. M. Nogueira (FARMEP, São Paulo, Brazil), respectively. The virus titers were 6.8 × 10^5^ PFU/mL, 1.44 × 10^11^ PFU/mL and 2.5 × 10^8^ PFU/mL for ZIKV, CHIKV and MAYV, respectively [9,10,29].

### 3.8. Cytotoxicity Assay

A portion of the ethanolic extract (10.0 mg) was dissolved in 1.0 mL of dimethyl sulfoxide (DMSO). This solution was used in the cytotoxicity assays, where Vero, LLC-MK2 and MRC-5 cell monolayers were trypsinized, washed with culture medium and plated in a 96-well flat-bottomed plate with 2.0 × 10^4^ cells per well. After 24 h of incubation, aliquots (8.0 μL) of the solution containing the extract were diluted in culture medium (DMEM, 1% FBS) at concentrations ranging from 400 to 0.78 μg/mL. An aliquot of 200 μL of each dilution was transferred to the wells containing the cell monolayer, and the plates were incubated for 48 h and 72 h for MRC-5 and Vero cells, respectively, at 37 °C in a humidified incubator with 5% CO_2_. The supernatants were removed from the wells, and 28 μL of MTT (solution 1 mg/mL in PBS, Merck^®^, Kenilworth, NJ, USA) was added to each well. The plates were incubated for 1.5 h at 37 °C, and DMSO (130 μL, Merck^®^, Kenilworth, NJ, USA) was added to the wells to dissolve the formazan crystals. The plates were placed on a shaker for 15 min, and the optical density was determined at 492 nm (OD_492_) on a multiwell spectrophotometer (Victor ^TM^ X3, Perkin Elmer^®^, Waltham, MA, USA). The results were obtained from four replicates, with at least four concentrations of each sample. Cytotoxicity was calculated using the equation (A − B)/A × 100, where A and B are the OD_492_ values of untreated and treated cells, respectively. The 50% cytotoxic concentration (CC_50_) of the assayed samples is defined as the concentration that reduces the OD_492_ value of treated uninfected cells to 50% of that of untreated uninfected cells [9,10,29,30].

### 3.9. Antiviral Assays

The Vero cell monolayer (2.0 × 10^4^ cells per well) was infected by viral suspensions with titers of 1.0 × 10^3^ TCID_50_/mL, 1.0 × 10^7^ TCID_50_/mL and 1.0 × 10^5^ TCID_50_/mL for ZIKV, CHIKV and MAYV, respectively [9,10,29].

The antiviral activity of the extract was evaluated using an MTT colorimetric assay [9,29,30]. Vero cell monolayers were grown in 96-well microtiter plates. Dilutions of the extracts and compounds in non-cytotoxic concentrations (100.0 to 0.78 μg/mL) were added to the wells simultaneously with the viral infection. The plates were incubated at 37 ºC in a humidified 5% CO_2_ atmosphere for a period of 72 h. Controls consisted of untreated infected, treated non-infected and untreated non-infected cells. Positive controls (ribavirin, PanVel^®^ (200.0 to 1.5 μg/mL) (Rio Grande do Sul, Brazil), amantadine, Eurofarma^®^ (100.0 to 0.78 μg/mL) (São Paulo, Brazil) and α-2a interferon, Bergamo, Brazil (4.0 × 10^4^ to 3.0 × 10^2^ UI/mL)) were also employed in each assay. In all experiments, toxicity control was performed (cells treated with the extract and not infected with virus). Cell viability was evaluated by the MTT colorimetric method, as described above for the cytotoxicity assay [9,29].

The 50% cytotoxic concentration (CC_50_) of the assayed samples is defined as the concentration that reduces the OD_492_ value of treated uninfected cells to 50% of that of untreated uninfected cells. The 50% antiviral effective concentration (EC_50_) is expressed as the concentration that achieves 50% protection of treated infected cells from virus cytopathic effect. The percentage of protection is estimated by equation [(A − B)/(C − B)] × 100, where A, B and C are the OD_492_ values of treated infected, untreated infected and untreated uninfected cells, respectively [9,10,29,30].

The CC_50_ and EC_50_ values for each sample were obtained from dose–effect curves. The CC_50_ and EC_50_ values are the averages of four assays carried out with eight different concentrations within the inhibitory range of the samples for Vero cells. The therapeutic index (i.e., selective index, SI) is defined as CC_50_/EC_50_ [31].

### 3.10. Statistical Analyses

Statistical calculations for the cytotoxic and antiviral MTT assays were performed with the GraphPad Prism 5.0 software package (Statistica). Results are expressed as the mean ± SD of three independent experiments. Student’s t-test was used for the statistical analyses; *p*-values > 0.05 were considered to be significant.

## 4. Conclusions

The leaves of *F. chica* are rich in flavonoids, with 4.09 ± 0.34 mg/g of dry plant material. Thirty-nine flavonoids were identified in the ethanolic extract in this study, highlighting the group of anthocyanidins widely described in the species and a series of flavones and their glycosides that are being reported for the first time in the species. The ethanolic extract of the leaves proved to be a potential source of antiviral compounds. Inhibition of the multiplication cycle of DENV 2, MAYV and ZIKV viruses was observed in in vitro assays. Flavonoids have a wide range of biological activities and may contribute to the observed antiviral activity.

## Figures and Tables

**Table 1:** Flavonoids partially identified in extract of *Fridericia chica* by LC-DAD-MS.

	Compound	Molecular Formula	RT (min)	UV (nm)	HRMS [M + H]^+^ (*m*/*z*)	Error ppm	[M – H]^−^ (*m*/*z*)	Aglycone (Mol. Wt.)	Characteristic *m*/*z* of Ions in Positive Ion Mode (%)
1	Vicenin II	C_27_H_30_O_15_	11.7	281, 330	595.1646	2.9	593.48	-	595.1646 (100.0), 577.1546 (27.0), 475.1221, 457.1122 (36.8), 439.1019 (20.5), 355.0807, 337.0700 (54.5), 295.0598 (30.8)
2	6-Methoxyluteolin-*O*-glucoside	C_22_H_22_O_12_	11.9	278, 330	479.1173	3.2	477.31	316	479.1173 (21.0), 317.0649 (100.0), 303.0454 (6.6), 302.0415 (49.1)
3	6-Methoxyluteolin-*O*-glucuronide	C_22_H_20_O_13_	12.1	283, 328	493.0962	4.0	491.55	316	493.0962 (34.0), 317.0648 (100.0), 303.0447 (9.7), 302.0416 (56.3)
4	Scutellarein-*O*-glucuronide	C_21_H_18_O_12_	12.4	283, 330	463.0881	1.1	461.43	286	463.1219 (1.1), 287.0907 (100.0)
5	Hispidulin-*O*-glucoside	C_22_H_22_O_11_	12.7	282, 333	463.1227	2.8	461.30	300	463.1227 (16.4), 301.0701 (100.0), 286.0467 (64.7)
6	6-Hydroxyluteolin-*O*-glucoside	C_21_H_20_O_12_	12.9	280, 333	465.1019	3.0	463.46	302	465.1020 (100.0), 303.0494 (4.3)
7	Hispidulin-*O*-glucuronide	C_22_H_20_O_12_	12.9	278, 330	477.1019	3.0	475.35	300	477.1021 (32.7), 301.0702 (100.0), 287.0502 (11.7), 286.0466 (79.0)
8	6-Hydroxyluteolin-*O*-glucuronide	C_21_H_18_O_13_	13.1	283, 330	479.0811	3.0	477.38	302	479.0811 (19.6), 303.0493 (100.0)
9	Scutellarein-*O*-rutinoside	C_27_H_30_O_15_	13.7	276, 335	595.1644	3.9	593.48	286	595.1649 (15.1), 287.0545 (100.0)
10	Scutellarein-*O*-glucoside	C_21_H_20_O_11_	13.9	277, 335	449.1068	3.2	447.39	286	449.1071 (10.4), 287.0545 (100.0)
11	6-Methoxyluteolin	C_16_H_12_O_7_	14.1	281, 335	317.0649	3.8	317.45	316	317.0650 (99.3), 303.0448 (15.4), 302.0416 (100.0)
12	Scutellarein-*O*-glucuronide	C_21_H_18_O_12_	14.1	281, 335	463.0860	3.5	461.24	286	463.0864 (20.5), 287.0545 (100.0)
13	6-Hydroxyluteolin-*O*-glucuronide	C_21_H_18_O_13_	14.2	283, 331	479.0809	3.2	477.31	302	479.0809 (19.6), 303.0494 (100.0)
14	Scutellarein-*O*-glucuronide	C_21_H_18_O_12_	14.3	276, 335	463.0861	3.1	461.50	286	463.0864 (22.0), 287.0545 (100.0)
15	3′-hydroxy-carajurone	C_16_H_13_O_6_^+^	14.5		301.0708	1.3		301	301.0707 (100.0), 287.0512 (14.0), 286.0472 (91.2), 186.0162 (20.6), 168.0056 (20.5), 140.0107 (11.4)
16	Apigenin-*O*-rutinoside	C_27_H_30_O_14_	14.7		579.1694	3.3	577.21	270	579.1699 (18.0), 433.1115 (15.6), 271.0594 (100.0)
17	6-Hydroxyluteolin	C_15_H_10_O_7_	14.9	277, 335	303.0493	3.6	301.36	302	303.0492 (100.0), 169.0132 (5.1), 135.0431 (3.8)
18	Apigenin-*O*-glucoside	C_21_H_20_O_10_	15.0	276, 333	433.1119	3.5	431.31	270	433.1119 (13.9), 271.0594 (100.0)
19	Carajurone	C_16_H_13_O_5_^+^	15.1		285.0761	0.7		285	285.0760 (100.0), 271.0565 (17.2), 270.0526 (94.5), 242.0574 (8.5), 168.0572 (12.3), 157.0652 (9.1), 144.0571 (9.0)
20	Hispidulin	C_16_ H_12_O_6_	15.3	276, 327	301.0699	4.3	299.27	300	301.0701 (100.0), 287.0499 (13.6), 286.0465 (98.7), 168.0050 (14.3), 140.0100 (10.5)
21	7-*O*-Methylluteolin-*O*-glucoside	C_22_H_22_O_11_	15.5	279, 335	463.1229	2.4	461.24	300	463.1229 (14.3), 301.0700 (100.0), 286.0469 (49.3), 168.0049 (2.9)
22	Apigenin-*O*-glucuronide	C_21_H_18_O_11_	15.5	268, 330	447.0914	3.0	445.30	270	447.0914 (21.4), 271.0596 (100.0), 153.0177 (4.0)
23	Scutellarein-*O*-(6’’-*O*-caffeoyl)-glucopyranoside	C_27_H_30_O_16_	15.5	283, 328	611.1380	3.4	609.29	286	611.1381 (18.4), 287.0545 (100.0), 163.0389 (11.1)
* 24	**6-Hydroxyluteolin-*O*-(6” -*O*-feruloyl)-glucopyranoside**	**C_27_H_28_O_18_**	**15.7**	**283, 330**	**641.1480**	**4.0**	**639.54**	**302**	**641.1488 (17.8), 303.0493 (100.0), 177.0540 (14.4)**
25	7-*O*-Methylluteolin-*O*-glucuronide	C_22_H_20_O_12_	15.9	-	477.1017	3.3	475.28	300	477.1018 (40.2), 301.0701 (100.0), 287.0538 (70.8), 286.0466 (58.2)
* 26	***O*-Acethyl-scutellarein-*O*-ramnosylgalloyl 3,5-dimethyl ether**	**C_32_H_30_O_15_**	**16.0**	**278, 335**	**655.1643**	**3.0**	**653.39**	**286**	**655.1646 (16.4), 287.0545 (100.0), 207.0649 (14.0)**
27	Scutellarein	C_15_H_10_ O_6_	16.4	284, 333	287.0543	4.2	285.16	286	287.0544 (100.0), 169.0129 (5.2)
28	Luteolin-*O*-(6’’-feruloyl)-glucopyranoside	C_31_H_28_O_14_	16.6		625.1536	3.4	623.36	286	625.1541 (15.4), 287.0544 (100.0), 177.0543 (11.2)
29	Luteolin-*O*-(6’’-*O-E-p*-coumaroyl)-glucopyranoside	C_30_H_26_O_13_	16.8	283, 328	595.1433	3.2	593.48	286	595.1437 (15.0), 287.0545 (100.0), 147.0435 (8.1)
30	Luteolin -*O*-(6’’-acetyl)-glucopyranoside	C_23_H_22_O_12_	16.8	276, 327	491.1173	3.2	491.36	286	491.1176 (15.1), 287.0545 (100.0)
31	Luteolin-*O*-(6’’-*O-E-p*-coumaroyl)-glucopyranoside	C_30_H_26_O_13_	17.7	283, 332	595.1431	3.5	593.35	286	595.1435 (27.6), 287.0545 (100.0), 163.0392 (12,0), 147.0438 28.8)
32	3′-hydroxy-carajurin	C_17_H_15_O_6_^+^	17.9		315.0865	1.0		315	315.0867 (100.0), 301.0670 (15.1), 300.0633 (86.8), 186.0161 (28.7), 168.0057 (17.2), 140.0108 (10.1)
33	Luteolin-*O*-(6’’-feruloyl)-glucopyranoside	C_31_H_28_O_14_	18.4	283, 335	625.1536	3.4	623.40	286	625.1539 (17.6), 339.1063 (12.5), 287.0544 (100.0), 177.0540 (20.9)
34	Luteolin	C_15_H_10_O_6_	18.5	270, 334	287.0543	4.2	285.16	286	287.0544 (100.0), 153.0179 (11.0)
35	Apigenin-*O*-(6’’-feruloyl)-glucopyranoside	C_31_H_28_O_13_	18.6	282, 330	609.1587	3.4	607.20	270	609.1592 (20.4), 271.0596 (100.0), 177.0543 (25.6)
36	Apigenin-*O*-(6’’-*O-p*-coumaroyl)-glucopyranoside	C_30_H_26_O_12_	18.7		579.1479	4.0	577.60	270	579.1488 (16.9), 271.0595 (100.0), 147.0437 (19.2)
37	Apigenin	C_15_H_10_O_5_	19.3	275, 331	271.0595	4.0	269.22	270	271.0596 (100.0), 153.0179 (12.9), 119.0487 (1.3)
38	Carajurin	C_17_H_15_O_5_^+^	19.4		299.0916	1.0		299	299.0918 (100.0), 285.0720 (19.6), 284.0686 (82.7), 269.0455 (17.9), 241.0503 (13.3), 169.0136 (13.6)
39	7-*O*-Methylluteolin	C_16_H_12_O_6_	19.6	275, 332	301.0699	4.3	299.40	300	301.0700 (100.0), 286.0465 (86.0), 168.0049 (10.0)

* New compounds.

**Table 2:** Cytotoxic activity (CC_50_) and evaluation of antiviral activity (EC_50_) against DENV 2, ZIKV, CHIKV and MAYV, in Vero cell line, with respective standard deviations (*n* = 3) and selectivity indexes (SI), for *F. chica* extract, performed by MTT colorimetric assay.

Extract Compound	MRC-5 CC_50_ µg mL^−1^	LLCMK_2_ CC_50_ µg mL^−1^	VERO CC_50_ µg mL^−1^	ZIKV CE_50_ µg mL^−1^	IS	CHIKV CE_50_ µg mL^−1^	IS	MAYV CE_50_ µg mL^−1^	IS	DENV 2 CE_50_µg mL^−1^	IS
*F. chica*—leaves	296.7 ± 1.4	370.2 ± 10.7	>400	40.9 ± 2.3	>9.8	NA	-	30.1 ± 2.0	>13.3	39.6 ± 1.1	9.3
Ribavirin (positive control)	NT	NT	370.4 ± 1.2 (1516.7 ± 1.2 µM)	94.47 ± 2.70 (386.84 ± 2.70 µM)	3.9	NA	–	29.21 ± 1.4 (119.61 ± 1.39 µM)	12.7	NT	–
Amantadin (positive control)	NT	NT	84.9 ± 1.2 (561.3 ± 1.2 µM)	NT	–	63.37 ± 1.70 (419.0 ± 1.7 µM)	1.34	NT	–	NT	–
Interferon α (positive control)	NT	NT	NT	NT	–	NT	–	NT	–	^a,b^ 2.5 × 10^3^	–

Note: NA: not active; SI (selectivity index): CC_50 (Vero)_/EC_50 (Vero)_; –: not determinate; NT: not tested; ^a^: 80 to 100% inhibition of cytopathic effect; ^b^: UI/mL concentration.

## Data Availability

Not applicable.

## Sample Availability

Sample of the compounds are not available from authors.
